# Importance of building a digital species index (spindex) for entomology collections: A case study, results and recommendations

**DOI:** 10.3897/BDJ.8.e58310

**Published:** 2020-12-23

**Authors:** Stephen C. Mason, Jr., Isabelle S. Betancourt, Jon K. Gelhaus

**Affiliations:** 1 Academy of Natural Sciences of Drexel University, Philadelphia, United States of America Academy of Natural Sciences of Drexel University Philadelphia United States of America; 2 Drexel University, Philadelphia, PA, United States of America Drexel University Philadelphia, PA United States of America

**Keywords:** species index, curation, collections, collection management, natural history, entomology

## Abstract

The Entomology Collection at the Academy of Natural Sciences of Drexel University (ANSP) contains approximately four million insect specimens including some of the oldest in the Western Hemisphere. Like most large entomology collections, no complete inventory of the species represented in the collection was available and even a physical search for a species could not ensure that all available specimens would be recovered for study. Between 2010 and 2014, we created a species-level index (called here spindex) of all species and their specimen counts at ANSP, along with each species’ location in the collection. Additional data captured during the project included the higher level classification of each species and type of specimen preparation. The spindex is searchable online: http://symbiont.ansp.org/entomology/. The spindex project documented 96,126 species in the ANSP Entomology Collection, representing about 10% of the described insect fauna. Additionally, over 900 putative primary types were discovered outside the Primary Type Collection. The completion of this project has improved access to the collection by enabling scientists and other users worldwide to search these collection holdings remotely and has facilitated staff in curation, research, collection management and funding proposals. A spindex is an important tool that is overlooked for planning and carrying out specimen level digitisation. This project is a case study for building a species-level index. A detailed protocol is provided, along with recommendations for other collections, including cost estimates and strategies for tracking progress and avoiding common obstacles.

## Introduction

Biological collections preserved in natural history museums are of immense value to science and society and harbour massive amounts of records of life on Earth (Bakker et al. 2020, Shirey 2018). As archives of the planet’s past, such collections are essential, not only for the study of taxonomy, but indeed for all biology (Winston 2007, Page et al. 2015). Entomological collections are amongst the largest of the biological collections (Short et al. 2018). To serve the entomological community effectively, insect collections are kept in an organised, curated state so that specimens are accessible for research. Entomology collections are partitioned, based on the specimen preservation method, since each method has different archival needs. For example, specimens preserved wet in alcohol have storage needs that are different from specimens that are stored dry on pins. Within preservation partitions, specimens are curated taxonomically and, within that, sometimes specimens are organised alphabetically or by locality. Other groups of specimens are separated because they have a special Type status, they are part of a particular research project or because they are of historic importance. Therefore, locating specimens can be complicated even in a well-curated collection because specimens of a given species may be in multiple locations.

Determining species holdings and locations within a collection has traditionally involved a physical search, which gives ample opportunity to overlook specimens. Today, computer technology and online networks have opened up powerful opportunities for more effectively managing collections and sharing collection information through the digitisation of specimens and their data. Digitisation is the process of making electronic copies of hard copy materials (Fabunmi et al. 2006). Many insect collections are actively digitising parts of their collections, which is making specimens and their data more accessible and increasing their impact (Short et al. 2018, Beaman and Cellinese 2012, Vollmar et al. 2010). The number of publications using digitised specimen information has steadily increased year-to-year and digitised collection information has been used for projects within the fields of systematics, taxonomy, evolution, biogeography and biodiversity assessment, but also invasive species, climate change, food and farming and species conservation (Short et al. 2018, Seltmann et al. 2017, Suarez and Tsutsui 2004). Thus, creating a digital species index that includes the physical location of the species within the collection can better serve the goals of collection retrieval, data accessibility and, ultimately, the progression of research.

The Entomology Collection at the Academy of Natural Sciences of Drexel University (ANSP), located in Philadelphia, Pennsylvania, USA, is a model collection to create a complete digital species index because of the size, age and organisation of the collection. At approximately four million specimens, its size ranks amongst the top 25 collections in North America (Arnett Jr et al. 1993, Cobb et al. 2019), so size is a challenge, but not insurmountable for collection-wide projects (5 years or less). Founded in 1812, ANSP is the oldest continuously operating natural history museum in the Americas (Peck et al. 2012) and the Entomology Collection holds specimens ranging from the 1820s to the present. The age of the collection presented varied states of curation as well. These included problems with outdated nomenclature, suboptimal organisation and misplaced specimens, making searches difficult for fulfilling loan and information requests, re-integrating loans and general curation. In addition, the lack of digital access to the collection holdings for users outside the physical collection was an obstacle in an increasingly digital world.

The goal of this digitisation project was to develop a digital species index (hereafter spindex) of all the curated specimens in the ANSP Entomology Collection and to map locations for all the specimens of each species to increase access to the data in the collection. In addition to the species present in the collection, goals were also to capture the higher taxonomy of each species, specimen preparation, number of specimens and type status of the specimens. A web-accessible search portal, where both ANSP staff and the public could search the collection holdings online at any time and from anywhere, was an important project goal. Finally, since we did not readily know of already-completed species inventories with these characteristics, we wanted to more thoroughly document how many of these projects were available for entomological collections in the United States and Canada. We predicted that, when this project was completed, the spindex would facilitate workflow within the department, prevent specimens from being “lost” or irretrievable within the collection and provide specific data in timely responses to enquiries from the entomological community.

## Methods

### About the collection

**Figure 1.**
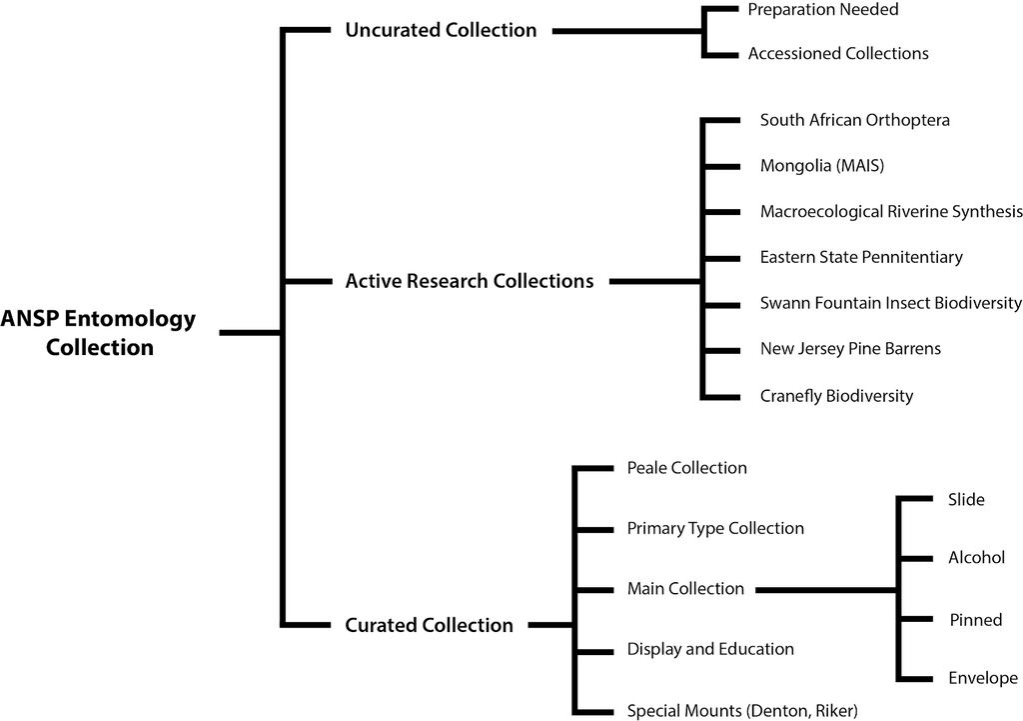
A schematic of the organisation of the Entomology Collection at the Academy of Natural Sciences of Drexel University.

The ANSP Entomology Collection (Fig. 1) holds one of the oldest, larger and more taxonomically-complete entomological collections in North America. The origin of the ANSP Entomology Collection is inseparable from the collection of the American Entomological Society (AES), a historic organisation whose active members included some of America’s most prominent 19th century systematic entomologists, such as James Brackenridge Clemens, Ezra T. Cresson, Sr., George Horn and John L. LeConte (Arnett Jr et al. 1993, Rehn 1959). The collections of ANSP and AES were combined in 1915. The ANSP Entomology Collection houses the Titian Peale Butterfly and Moth Collection, which is one of the oldest insect collections in North America, with specimens dating to the late 1820s (Gelhaus and Clark 2012, Gelhaus et al. 2004). The Primary Type Collection is exceptionally large with nearly 12,000 species, whose descriptions span the entire history of the collection (Shirey 2018).

Beginning in 1900 with the arrival of James A. G. Rehn (Gurney 1965) to the present tenure of Curator (now Emeritus) Daniel Otte, there has been an unbroken chain of Orthoptera curators and researchers at the Academy, making the holdings of the orthopteroid orders, Orthoptera, Phasmida, Blattodea, Dermaptera and Mantodea rank as some of the most comprehensive in the world. Rehn and his collaborator, Morgan Hebard, added hundreds of thousands of orthopterous specimens to the Academy’s collection during the first half of the 1900s. These holdings have been strengthened by the additions of the historic Lawrence Bruner and Samuel H. Scudder Orthoptera Collections and the extensive specimen and associated sound recording collections from North America, Africa, the Caribbean and Hawaii made by Otte.

The collection has grown, as well as in other groups of insects. Remarkable contributions from early to mid-20th century systematists, including Ezra T. Cresson Jr. (Diptera), Selvyn Roback (Diptera), Philip Calvert (Odonata), Annette F. Braun (Lepidoptera) and Emlen P. Darlington (Lepidoptera), have grown and shaped the collection (Arnett Jr et al. 1993). The aquatic insect collections, in addition to the major groups above, include important vouchers from over fifty years of stream and river surveys (Rosenberg 2009). More recent additions have been made by the current curator Jon K. Gelhaus (Diptera) from his research activities in North America and Mongolia (Gelhaus 2012). Recent accessions of collections, such as the Frank Fee Collection (Diptera, Coleoptera) (Gelhaus and Weintraub 2016), Alan G. Goodridge (Lepidoptera), H.T. Enterline (Lepidoptera), R.T. Allen (Non-insect Hexapods) and from Rutgers University, Stockton University, University of Delaware and the University of West Virginia have continued to grow the collection substantially.

### Template file

We started the species level inventory by creating a template in Microsoft Excel for data entry. Microsoft Excel was selected for the initial data input because it is commonly available, familiar to most data compilers and a simple application for bulk entry of tabular work. The template file included fourteen data entry fields: UNIQUE NUMBER, INPUT BY, ORDER, FAMILY, GENUS, SPECIES, SUBSPECIES, AUTHOR, NUMBER OF SPECIMENS, PARATYPES, SEE TYPE COLLECTION, PRIMARY TYPE?, CURATION and COMMENTS (Table 1). Many of these fields match the Darwin Core Standards to facilitate the sharing of information about biological diversity (Wieczorek et al. 2012). The nomenclature fields were entered verbatim according to the unit tray header labels or by species determination (identification) labels on the specimens. Our protocol specified that every field had to be populated to demonstrate that no field was missed during data input. We defined the populating of these species inventory fields as *spindexing* and the person spindexing as a *spindexer*.

### Workflow

**Figure 2.**
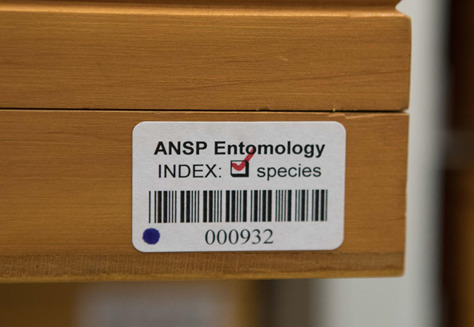
Each unique identifier placed on an ANSP Drawer (container) has a human-readable number and a barcode. The red check mark indicates that the contents have been spindexed.

**Figure 3.**
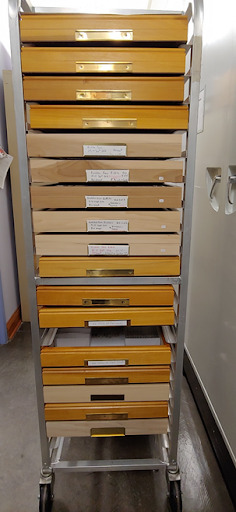
Prior to spindexing, a retrofitted food-tray cart was filled with 20-40 ANSP drawers to bring to a work space for labelling the drawers with the unique identifier. This method kept the aisles of compactorised storage cabinets free for others to use.

Each container in the Main Collection went through the spindexing process. We use “container” as a general term for anything that stores insect specimens, for example, wood and glass-topped specimen storage drawers (ANSP Drawer), vial racks, slide boxes, Odonata envelope boxes and wooden Schmitt boxes. Each spindexer placed a unique identifier label, which has a human-readable unique identifying number (Fig. 2), on each of the containers in a queue to be spindexed. Although we experimented with data capture in the collection aisles, we found it most efficient to place 20-40 containers on a retrofitted food-tray cart to bring to a desk for processing (Fig. 3). This system kept the collection space open so that others could access the different aisles, even when active data input was occurring. Once all the appropriate data from a container were entered into the spindex Excel template, a red check mark was placed on the unique identifier label above the unique number to show that the container had been spindexed (Fig. 2). At the same time, every container containing pinned specimens was checked for active dermestid beetle damage and infestations and cycled through a -20^0^C freezer in the carts. At the end of a data collection session, each spindexer saved their file on the server.

### Monthly review and progress tracking

At the end of every month, all the Excel files from the spindexers were stitched together to create a “master list” to check monthly activity statistics, such as numbers of specimens, species and containers spindexed. This also was a way to save the data and correct any inconsistencies and typographical errors within a single aggregated file. Aggregating data like this made editing easier. It particularly helped catch obvious misspellings and other types of human error. After editing, the monthly spindex data records were uploaded and maintained as a relational database implemented into FileMaker® Pro (FMP). The FMP format of these records facilitated searching, filtering, correcting and quality checking the accumulated data throughout the project. Another advantage of FMP was that it moved data to an online search portal efficiently.

The monthly review also allowed project managers to track individual and total progress, i.e. whether the project was slowing down, staying consistent or ahead of schedule. Since the total number of specimens and species could not be easily counted at the beginning of the project, we counted the number of initial containers and then used the number of containers spindexed to measure progress and keep us on schedule.

### Previously databased collections

The ANSP Entomology Collection contains three relatively-large collections that were digitised at the specimen level prior to the spindex project. These are the historic Titian Ramsey Peale Butterfly and Moth Collection, the Primary Type Collection and the Stream Survey Project. The Peale Collection, designated by the federal government as one of “America’s Treasures” (Gelhaus et al. 2004), had received funding to conserve and document its unique storage boxes and identified specimens. It also captured specimen level data and imaged each specimen. The Type Collection received National Science Foundation (NSF) funding for re-housing and for creating a digital specimen level database. The Stream Survey digitisation project, also NSF funded, involved freshwater organisms, including thousands of insects and included specimen-level data capture, re-curation and re-labelling. For each of these three collections, the digitised databases are available online and contain all the label data for each specimen.

The pre-existing data from the Peale and Primary Type Collections were used to populate the spindex template. The fields that still needed to be filled in to complete the spindex template entries were the UNIQUE NUMBER and the NUMBER OF SPECIMENS. Although the Primary Type Collection is mostly single holotype specimens, there were series of cotypes, syntypes and occasional allotypes and paratypes that needed to be included. An additional benefit to spindexing each container in the Primary Type Collection was that the accuracy of the previous database was checked and updated.

### Data-input problems and solutions

As a counterpart to this project, each container of pinned insects was placed in the freezer for preventative pest control. The first problem we encountered was that the initial batch of the adhesive paper, unique identifier labels were falling off the varnished ANSP-style insect storage drawers particularly when damp from condensation as they emerged from the freezer. We began using Gaylord's Spine Laser/Inkjet Foil-back labels, specifically designed to stay on the wood, varnished drawers. The increased durability of the foil-back labels had another benefit, which enabled us to peel the labels off deliberately, if needed, to be re-attached to a different container. We encountered alcohol vials that were filled with hundreds of specimens. Capturing the exact number of specimens in these vials would have been intensive and time-consuming and was not necessary to accomplish the main goals of this project. We decided that, if a vial contained fewer than ten specimens, each individual was counted. For vials with more than ten specimens, a rough estimation was made by multiples of ten.

Data were reviewed weekly to find inconsistencies, which often were a result of spindexers responding to data entry issues in different ways. For example, a commonly-encountered issue was finding a labelled unit tray without any specimens. Specimens may have been missing from a unit tray because they had been part of a long-term specimen loan (without a note indicating the loan) or because they had been misplaced. In some cases, such as in our Coleoptera collection, it appeared that the tray was a long-ago inserted placeholder for future specimens of this taxon (often noted by a lack of any pinholes in the hard-bottomed tray). In all cases, we left the unit tray in the drawer, but found two common ways spindexers handled the empty unit tray: a value in NUMBER OF SPECIMENS recorded as “0” or left blank, implying there were zero specimens. However, leaving a cell blank could also indicate that it was accidently skipped over. Thus, filling in all the NUMBER OF SPECIMENS cells with a value was added to the protocol.

As in many entomological collections, there are often many specimens identified only to the family level, usually at the end of the curated series for that family. In these cases, specimens determined to family level only were entered as FAMILY <name>, then GENUS as “undetermined” and SPECIES as “sp.” or “spp.” Another interesting data capture challenge included a surprising number of specimens with labels indicating the species was new to science, but was not clearly associated with a researcher’s name or specific epithet. These specimens were entered into the SPECIES field as “n.sp.” as they might be of particular interest to future researchers.

### Curatorial decisions

**Figure 4.**
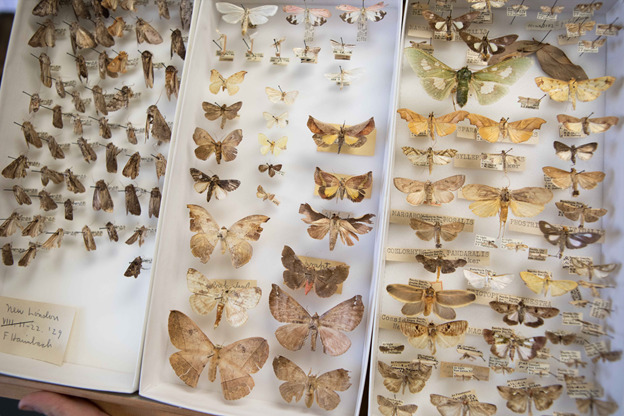
Example of a “catch-all” drawer with potentially dozens of different species. An effort was made to integrate specimens to their proper locations in the curated Main Collection before spindexing occurred.

Although the spindexing can handle the species, specimens and drawers wherever they are encountered, we found it to be more efficient to scout ahead and do some curation of the collection pre-spindex. Having each species grouped together is more efficient for the spindexing process as it results in less species entry lines. It also saves time with future maintenance of the spindex database because spindexing material that is out of place necessitates revisiting the database at a later time when the material is placed in its proper curated position. Curating and spindexing is a two-step process, while spindexing, curating and then updating the spindex after the curation is a less efficient three-step process. Curating takes time, so we selectively chose when to curate before spindexing to keep the project on schedule. In some cases, substantial curatorial reorganisation was needed before spindexing. For example, specimens on microscope slides were housed in different parts of the collection in a diversity of storage containers. A single species or genus was housed in separate slide boxes, often leaving the storage box mostly empty, resulting in the entire Slide Collection taking up considerable storage space. Since the Slide Collection was not under active research, we decided to consolidate it during the spindexing process. The slides were rehoused into Eberbach cabinets with horizontal pull-out metal trays. In the new slide container system, the slides were in a continuous series of trays that had no space for labels, contrasting with the previous containers which primarily consisted of hundreds of small labelled boxes. Therefore, we attached charts indicating the species arrangement to the outside of each cabinet to assist with the retrieval of needed taxa. Sections of the collection that needed curatorial attention prior to spindexing included large loan returns, donated collections, groups of unsorted (= unidentified to any classification level) specimens and catch-all drawers, some containing hundreds of determined, though misplaced, species (Fig. 4). In these cases, we prioritised specimen curation. In an effort to keep the project on schedule, only the higher priority sections were curated and spindexed within the project window. There was also a considerable amount of unprocessed material in the Main Collection spaces that we placed into a *pro tem* space. The protocol was to remove this material to a *pro tem* area until such material had been processed and sufficiently prepared to integrate into the Main Collection. In addition, specimens in the Main Collection that were not determined to at least family level were removed to *pro tem*. We grouped them in *pro tem* by order and as a full department activity, had a day where we sorted Diptera, another day for Coleoptera and finally a Lepidoptera sort to at least family level. Then, we integrated the sorted specimens back into the Main Collection and added them to the spindex.The size, age and limited staffing of the ANSP Entomology Collection meant that the nomenclature in most of the collection had not been kept updated. To maintain progress to ensure that we would complete the species index for the entire collection in the grant-funded period, we made an early decision to not update taxon names during the construction of the database [the KISS principle, Dalzell 2018]. The species names on the specimens and on the unit trays were entered verbatim. We decided this because worldwide catalogues are not available for many groups, either online or printed and checking for updating names requires time, expertise and subsequent re-labelling of storage containers. We determined that the updating of nomenclature and re-curation could best occur using our new digitised format after we completed the spindex. For example, we could then send a digital list of our species holdings to taxonomic specialists who would easily recognise outdated names and update these names. Our mantra was to finish the inventory even with outdated nomenclature (which are still searchable names), rather than not finish the project.

Another early decision we made to maintain efficient progress was not to attempt to locate any short or long-term specimen loans. Even though there are many specimens and species that may be absent from the spindex because they are on loan, tracking down that information is time-consuming, especially since our loan inventory system is not completely digital. The older loans involve searching paperwork and correspondence potentially spanning decades. The more recent loans usually have a note in the unit tray with loan information. For our protocol, the spindexer captured the data from any specimens left in that unit tray and typed in the COMMENTS cell that a certain amount of specimens were on loan.

### Species location database

We included in the database each container’s location in the Curated Collection. Mapping specimens would increase efficiency in locating specimens for collection management and research, an important goal of this project. Each aisle, each cabinet within an aisle and each column of containers within a cabinet was given a coordinate. The location code for the indexed specimen containers was created by combining the coordinates of the aisle, cabinet and column. Through this process, we created a map of the species’ locations in the collection. A Scanfob 3002i Bluetooth 2D Barcode Scanner was then used to scan the spindexed containers’ UNIQUE NUMBERS, for automatic entry into an Excel spreadsheet with the typed out location codes. We collected the location data when we finished spindexing the entire Curated Collection and it was integrated into the spindex using FMP. Thus, by looking up a species’ coordinates in our completed spindex database, its specific location(s) in the collection can easily be found.

We collected baseline data on the length of time it took to find 25 species located within the Curated Collection prior to this spindex project. Respondents, varying in expertise from volunteers and students to research associates and curatorial staff, completed the search metric test during late 2010 and 2011.

### Determining the prevalence of species indices

To document how many of the larger entomological collections (3.5 million specimens and greater) in the United States and Canada had a complete species inventory that was accessible online through a public web-accessible search portal, we first assembled a list of the 25 largest collections. We used Arnett Jr et al. 1993, Cobb et al. 2019 to create this list since they provide the approximate number of insect specimens in major North American insect collections. With the list developed, in June 2020, we checked each collection’s website and e-mailed a contact person with several questions to obtain information on the collection size, the presence and completeness of a collection-wide species inventory and if there were a searchable web portal for the species inventory. If we did not receive a response from a collection’s contact following two requests, we used the information available on the institution’s website and Cobb et al. (2019).

We followed up with collections that had a near-completed species inventory to see if they also had a species location database. We defined “complete” as having the ability to find nearly all the available specimens (> 90%) for a certain species in their collections. With this information, we were able to assess the novelty of the ANSP spindex and web portal and compare the characteristics of our inventory with those of other larger collections.

## Results

### The Spindex of the curated collection

**Figure 5.**
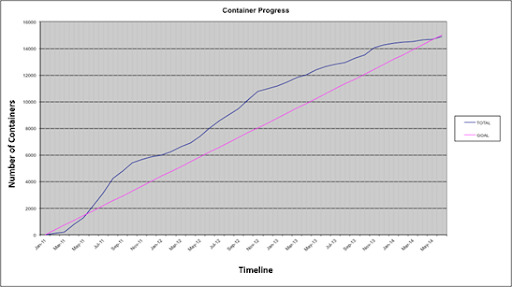
Cumulative numbers of spindexed containers over time. The pink line represents the number of containers needing to be spindexed to complete the spindex project by the deadline. The blue line represents the cumulative number of containers that were actually spindexed by month. After the initial start, the spindexing was ahead of the projected goals and completed by the project deadline.

**Figure 6.**
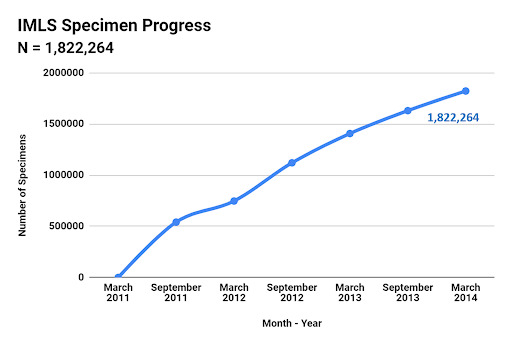
Progress in databasing specimens for the spindex project during the period of March 2011 through to the end of data capture in March 2014. The total number of identified specimens was 1,822,264 specimens.

**Figure 7.**
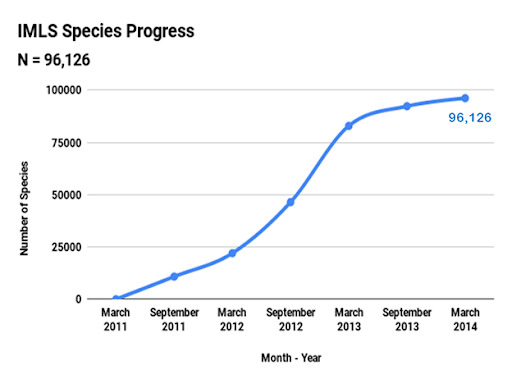
Progress in databasing species for the spindex project during the period of March 2011 through to the end of data capture in March 2014. The total number of species was 96,126 species, which represents approximately 10% of the world’s known insect fauna.

The Spindex project started in October 2010 and was completed in September of 2014, with the spindexing taking approximately three years. Two staff members managed and worked on the project full-time (for approximately 80 person-months). Across the course of the project, over twenty part-time staff, undergraduate students and volunteers contributed to spindexing as well as sorting, transferring and identifying specimens and completing other general curatorial tasks, prior to spindexing. By the end of spindexing, in March 2014, a total of 14,910 containers had been spindexed (Fig. 5), containing 1,822,264 *curated* specimens (Fig. 6) and 96,126 species (Fig. 7), nearly 10% of the world’s described diversity of insects (Grimaldi and Engel 2005). These specimens and species represent 80 orders and 1,060 families of invertebrates.

The cost for the completed project was $300,000, with half of this external support from the Institute of Museum and Library Sciences (IMLS) and a required matching half from internal funding sources of the Academy (Collection Care Upgrade Fund). This resulted in data capture costs of $0.16/specimen, $3.12/species and $20.12/container. Additional help came from our corps of volunteers, including unfunded student co-op positions and some project management costs covered by other internal funding sources (curator, collection manager) but these were not calculated in the total monetary cost for the project.

**Figure 8.**
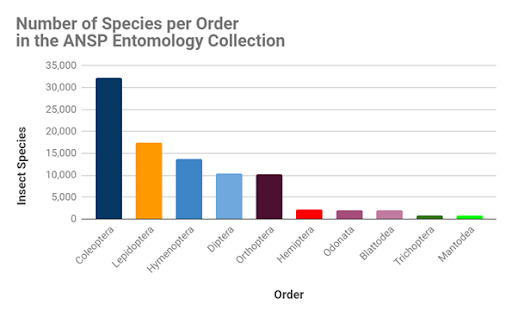
Number of species per order in the ANSP Entomology Collection. Even though Orthoptera is not part of the “big five” insect orders in terms of number of world species, it samples the order’s total richness greater than much more species-rich orders like Lepidoptera and Coleoptera.

**Figure 9.**
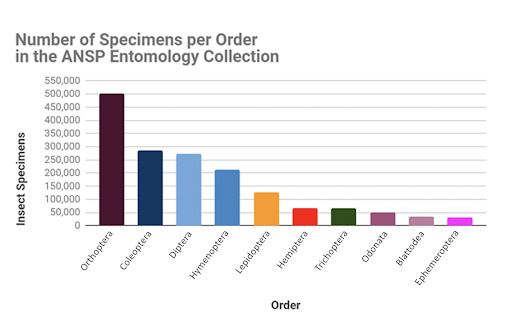
Number of specimens per order in the ANSP Entomology Collection. Orthoptera is the most abundant, which shows the strength of this group in the collection. Although the Coleoptera collection represents far more species in our collection than any other order, the number of specimens is approximately half that of Orthoptera and similar to that in Diptera, indicating an average of a few specimens per species.

The order Coleoptera had the highest species richness with 32,178 species, followed by Lepidoptera with 17,384 species, Hymenoptera with 13,716 species and Diptera with 10,335 species (Fig. 8). Orthoptera, our collection’s main strength, ranks fifth in species richness with 10,120 species and represents 35% of the world's recognised species (28,530, Cigliano et al. 2020). The order Orthoptera had the most specimens (500,620), approximately double the number of specimens in the second and third orders, Coleoptera (285,710) and Diptera (273,257), respectively (Fig. 9).

The commonest species epithet was “sp.” denoting the specimen was not identified to species, just to genus. The next most common species epithets in the collection are *bicolor*, *gracilis*, *affinis*, *elegans*, *occidentalis* and *californicus*/*a* (Table 2). We captured 284,229 spindexed specimens only determined to the family level and 16,472 spindexed specimens to the genus level. Of these, 449 entries, represented by 2,604 specimens, have a determination label, but the information was illegible (“unreadable”) and, therefore, inaccessible. Another 211 species’ entries are labelled as “n.sp.” representing potentially-undescribed species we have in our collection noted by former scientists (Table 2). The most common author surnames for described species in the collection are Cresson Sr. with 3,629, LeConte with 3,509 and Fabricius with 2,956 (Table 3) reflecting the importance of E.T. Cresson Sr. and J. LeConte in Philadelphia entomology, as well as in the origins of North American Hymenoptera and Coleoptera research, in general.

**Figure 10.**
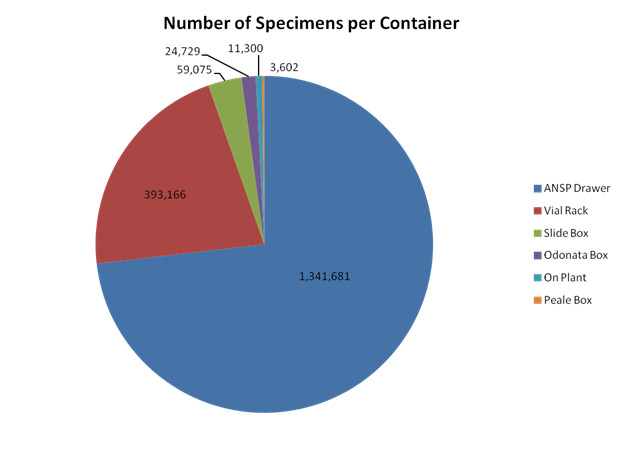
Total number of determined specimens curated by storage container. Nearly 75% of curated specimens are on pins and housed in ANSP-style drawers. Odonata Box refers to Odonata specimens preserved in envelopes. Specimens (mostly Coccoidea) that were preserved on their host plant are represented by “On Plant.” Peale Box refers to the historic specimen boxes housing the Peale Lepidoptera Collection.

Most of the specimens in the Curated Collection are preserved dry and pinned in ANSP drawers (1,341,681 specimens), followed by specimens stored in ethanol in vial (393,166 specimens) and then specimens prepared on microscope slides (59,075 specimens) (Fig. 10). Other specimens are stored dry in envelopes (24,729), *in situ* on plant material (11,300) and in the historic Peale Boxes (3,602 specimens).

By cross-referencing the Primary Type Collection with the spindex project, 1,016 primary types, comprising 1,406 specimens, were added to the updated Primary Type Collection Database, which resulted in a total of 11,709 species represented by primary types in the collection. By including the PRIMARY TYPE? field in the spindex template, over 900 putative primary types were found in the Main Collection and highlighted in the Species Index database. These specimens can now be researched to determine whether they actually are primary types to be added to the Primary Type Collection or unpublished (manuscript) primary types.

By working through the collection for spindexing, other important specimens were uncovered in the Curated Collection, such as specimens collected by Titian Ramsey Peale, which were not in his original, custom-designed boxes. Over 100 Peale specimens, represented by over a dozen species [including the extinct uraniid moth, *Urania sloanus* (Cramer, 1779)] were taken out of the Main Collection and placed and spindexed with the Peale Collection.

### Decreased species search time

Prior to the spindex, our baseline search metrics took an average of 13.55 minutes to find a species and one species was not found by any of the tested staff in the 30 minute time period allotted for each species search. Using the specimen coordinates that we included in our spindex, we are able to map out the location of specimens in the collection. Our searches now take less than a minute and the spindex includes all the locations for a species within the collection. For complicated searches (multiple species and locations), results can be printed if needed and taken into the physical collection to access the specimens.

### Web accessible search portal

An internet search portal for species in the ANSP Entomology Collection was created and is accessible to in-house staff, the public and researchers around the world: http://symbiont.ansp.org/entomology.

There are two different views on the website, an internal view and an external (public) view. The internal view is for entomological staff and displays the full set of collected data fields, which are all useful for collection management, including location within the collection. The external view displays fields that are relevant to the external users, such as species name, number of specimens and preparation type. When relevant, search results link to the Type and Peale Collection websites for a “one-stop shopping” experience when digitally searching the collection.

**Figure 11.**
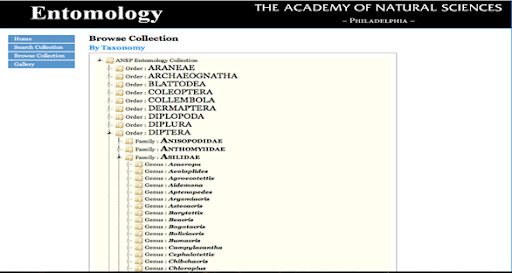
Species index (spindex) online database showing the “Browse” section. The taxonomy of a given group can easily be seen. In this example, families of true flies (Diptera) and genera within one family (Asilidae) are displayed.

**Figure 12.**
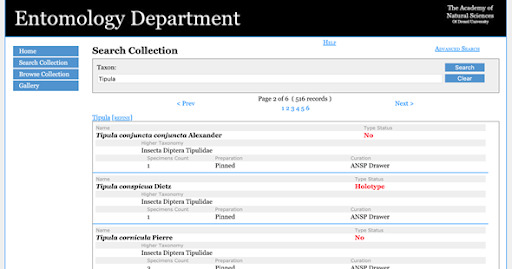
Species index (spindex) online database showing the “Search” section. By searching, the user can input the specific taxonomic name or part of the name to retrieve records for the pertinent group. In this example, the genus *Tipula* was searched and 516 records were recovered, including links to the Type Database with complete specimen label information (see *Tipula conspicua* entry).

The user can either “Browse” or “Search” the new database. When browsing, the user starts at a higher classification and moves to lower taxonomic levels (Fig. 11). Using the search function, the user inputs a specific taxonomic name or part of a name to retrieve records for the pertinent group (Fig. 12).

### Collections with a Complete Species Index and location database

Out of the 25 largest insect collections in Canada and the US, four had a complete species inventory: the University of Missouri, the Enns Entomology Museum (UMRM), the Natural History Museum of Los Angeles County (LACM), the University of California, Berkeley’s Essig Museum of Entomology (EMEC) and the Academy of Natural Sciences of Drexel University. The largest 25 collections ranged in size from 3.5 million to 35 million specimens and the four collections with completed indexes ranged in size from 4 million to 7 million specimens. Two of these four, LACM and ANSP, also had a complete location database mapping the location of species in the collection. EMEC reported having a partially-complete location database (Suppl. material 1). The 21 other insect collections reported having a partial species inventory. Of these, there was a large range in the completeness of the inventories that spanned from lists of type species to complete species lists for one or a few insect orders, as well as species lists that were a by-product from specimen-level databasing projects.

## Discussion

### Novelty and significance

Capturing species names of the millions of curated insect specimens originally seemed a daunting task, but turned out to be straightforward, although time-consuming, due to the size of the collection and the variety of specimen preparations and varying levels of curation. However, after four years of the spindex project, we are one of the the first major entomology collections in the United States and Canada to have a complete species-level inventory with an online search portal and one of only two collections that have a database that maps the specific location(s) of a given species in the collection.

As predicted, the spindex has reduced the time involved in locating species, facilitated collection management and has prevented specimens from being overlooked. To reiterate, this was a species-level inventory, not a specimen-level project. With the complete spindex, we have a greater understanding of how our collection is organised, including its strengths and weaknesses and we have the ability to provide quantifiable metrics.

Knowing which species and specimens the ANSP Entomology Collection houses and their locations and being able to advertise their availability for study, makes this collection an extremely important, but now *efficient* “library” of species for scientific reference. The species index, our first complete modern inventory, is a tool to allow the most effective use of the collection for scientific research. Entomology curatorial staff know the significance of complete collection inventories for responsible management, yet the large size of these collections and the cost of such projects make completing these inventories appear unobtainable. We hope that our work encourages and inspires them to start and *finish* their own spindex!

Our survey of the 25 largest entomological collections in the US and Canada showed that all the collections had made modest to substantial progress in digitisation of specimen data, but few have a complete digital species inventory. This is likely the influence of the funding available for these projects through the NSF Advancing Digitization of Biodiversity Collections (ADBC) programme which emphasises specimen-level data digitisation. In these cases, a species inventory slowly accumulates as a by-product of the specimen digitisation. This result, a partial species inventory, is usually skewed to the project(s) taxonomic emphasis and is, therefore, of limited utility. We chose a different route of funding, through the IMLS programme and emphasised the value of the species inventory as a necessary curatorial tool long overdue in entomological collections. It has also proven to be a valuable tool in all aspects of our collection work, particularly as a foundation for our specimen level digitisation projects (OrthopNet, LepNet) funded through NSF ADBC. We suggest that a species-level data digitisation inventory should be a high priority before or alongside substantial specimen-level digitisation efforts.

### Project management

Counting the number of specimen containers at the beginning of this project was essential for monitoring progress. Unless a collection has completed any kind of inventory, the number of specimens and species are only rough estimates. Counting the containers is a straightforward task that provides an exact count, not an estimate and that takes a short amount of time. The 14,910 containers in our collection provided a metric for tracking progress and also helped us determine when we had time for the complementary curatorial projects that happened in conjunction with spindexing.

Progress in spindexing appeared to be slowest when working with poorly-curated parts of the collection. This included unincorporated loan returns and donated collections, misplaced specimens and uncurated “catch-all” drawers that have an assortment of different species all together. Initial complementary projects that took a modest amount of effort curating these parts of the collection became a priority because consolidating species’ locations saved spindex processing time. For example, more than 10,000 specimens identified to genus and/or species from the Laurent and Endy (Coleoptera) Collections, which had been separate for decades, were finally integrated into the Main Collection. We also removed thousands of specimens that were undetermined from within the Main Collection. With the help of volunteers, students and staff, we had Ordinal “sorting events” that resulted in the determination of these specimens to the family level at least. Once at the family level, we could include the specimens into the Main Collection. Not only did the integration of these specimens pre-spindex streamline the spindexing process, it also added hundreds of new species and dozens of new genera to the Curated Collection spindex, making the collection more comprehensive and a better tool for science.

We were happy with the decision we made not to check and update the taxonomy of every species in our collection. This effort would not be complementary to the spindex project because of the large amount of time involved. Updating nomenclature does not affect the aggregation of species into the same curated location. Therefore, it was not a priority for the project because it was a task that could be completed after the spindexing. In fact, it is faster to update nomenclature post-spindexing. With the completion of the spindex, it is now feasible to send entire taxonomic lists from the collection to experts, who can quickly update nomenclature and authorship and correct misspellings. Experts can also find it helpful to have species lists of entomology collections for their own records. As with any tool, the spindex needs to be maintained. It is the responsibility of all curatorial staff to keep track of any specimens that are moved and names that are changed. As the Curated Collection has been completely spindexed and the location of every specimen of a species is known, it is important to keep this database current to maintain its accuracy. The spindex and the Curated Collection *need* to be a mirror image of each other. The spindex requires updating when any of the following common curatorial tasks occur: specimens are physically reorganised, specimens are added or taken away, nomenclature is updated or species determinations are changed. These activities might especially happen when integrating specimens re-determined from loan returns or also when additions to the collection involve an expansion into new containers. Updates can be made directly to the spindex, to paper forms or a combination of the two, as we have used. If using paper forms, updating the spindex must be done promptly to avoid the forms accumulating.

Researcher visits can involve changes in curation, ranging from simple to comprehensive. For simple tasks, such as a few re-determinations of species, we can update the spindex immediately. However, when a researcher carries out substantial updates to the curation of an insect group, we have found it more efficient to re-spindex that entire group instead of track individual specimens in a piecemeal fashion. When a visiting researcher to our underwing moth (Erebidae: *Catocala)* collection corrected numerous misidentifications, updated nomenclature and integrated additional specimens, the re-curated collection was substantially different from the spindex. Thus, we deleted the records for the specimen containers and re-spindexed them. Without continually updating the species index as curatorial changes occur, it becomes just a historical record of what the collection contained as of the project completion (in our case, 2014).

### Broader impacts

The spindex is able to provide easily-accessible collection data for grant opportunities. By being able to supply numerical data about species, specimens and type preservation, it helped to obtain funding for two NSF Thematic Collection Network (TCN) grant applications, LepNet and OrthopNet. For a proposal involving multiple North American collections of Odonata, we were able to provide information within minutes to the principal investigator (PI) that revealed that the Academy’s Odonata collection included forty species not represented in other major collections. Lastly, a multi-institutional proposal for digitising North American bee collections is in preparation as this paper is written. Due to the spindex being digital, we were able to supply the PIs with detailed collection statistics even when the current COVID-19 pandemic prevented us from physically entering the collection. With the completed spindex, we were able to save time, create less stress and were confident in the information we provided involving our complete Curated Collection, making every grant proposal stronger.

The online search portal allows external users to assess the completeness of a collection. The Academy carried out two major collection exchanges in the 1960s, one involving the exchange of part of the Lepidoptera from the ANSP Entomology Collection with the Orthoptera from the Carnegie Museum and another involving the exchange of part of the Coleoptera from the ANSP Entomology Collection with the Orthoptera from the Museum of Comparative Zoology. This led to misperceptions often heard from specialists that ANSP no longer had significant Coleoptera or Lepidoptera collections. The online portal allows researchers to browse the current holdings of these two Orders. Researchers soon realise that large collections in these two orders still remain at the Academy with over 30,000 species in Coleoptera and 15,000 species in Lepidoptera.

The online search portal has enabled researchers to preview the collection contents themselves. The first response to any researcher queries about the collection includes sending the link to the spindex to make sure they are aware of this tool. Knowing the extent of our taxon holdings, researchers can better plan their visits to the Academy and/or enquire about the possibility of borrowing specimens and specifically which ones. Furthermore, the search portal reveals the existence of specimens with multiple preparations.

Projects like this species inventory can provide valuable outreach opportunities. At two national conferences, the Lepidopterist’s Society 2014 annual meeting and the Entomological Collections Network 2016 annual meeting, we presented results of the spindex project to our target audience, the entomology community. Outreach has also occurred through social media, principally through *Instagram, Periscope* and *Twitter.* Examining the entire Main Collection during data capture provided opportunities to share interesting specimen findings and to highlight milestones of the project.

The spindex project also was important outreach for on-site activities. Tours of the Entomology Department have included demonstrations of the spindex project, with discussions of how the project has facilitated the department’s mission of providing specimens and data to the scientific community. The sixth annual *Bugfest* event was held in the public museum during the weekend of 10-11 August 2014 and the spindex project was introduced to approximately 3,000 visitors. Similarly, *Member's Night* in 2014 included over 1,000 attendees where we showcased the advantages of the completed project. All collection tour visitors can see that containers in the collection each have a unique identifier label with a red check mark on it and learn how the availability of this information through the spindex helps scientific researchers find the specimens they need. Visitors are always impressed to know the full and accurate scope of the collection size and its species representation, but shocked to realise that the collection only represents about 10% of the currently-described insect species! This can only have been demonstrated by the completion of the spindex.

### Recommendations

We provide here recommendations for other collections that are considering carrying out a species inventory as our project represented obstacles many collections will face.

- Estimating Costs. We believe our project provides realistic estimates using cost per container to predict the total budget for completing your spindex. In the case of better curated collections, the total budget might be less. Future projects might find it useful to keep track of all paid and unpaid personnel efforts.

- Track Progress. Set your goals and track the progress based on what can be measured. We found the number of containers was easy to count at the beginning of the project and was simple to use to track progress.

- Simplicity. Keep the captured data simple so that the collection can be completely inventoried *in the time allotted*. Data, such as geographic locations and condition of specimens, are useful, but can slow down the project and is not the priority.

- Adjust Goals and Strategies. Keep the overall goal(s) in mind and adjust as needed when problems arise. A completed spindex should be the main priority and problem-solving should keep that priority paramount.

- Curation – now or later? Think strategically about only re-curating parts of the collection when it can actually save time completing the spindex. The completed inventory is the goal, not a fully re-curated collection.

- Avoid Side Projects. Defer major projects that can be expedited with a completed spindex until the end of the project, such as updating nomenclature.

- Spindex Accessibility. An easily found online portal for searching your spindex should be the first place a user can go to query your collection. It helps if the portal allows “one-stop shopping” for all available collection information.

- Updating is Essential: Set up a workflow that all collection staff will follow to update the spindex. Do not allow the spindex to become a historical record of your collection.

- Use the Spindex. You now have an important tool in collection management, for daily curation tasks, in preparing grant proposals, selecting material for outreach and other uses you will soon discover.

- Intimacy with your Collection: A spindex provides the opportunity for current staff to examine every part of the collection, by pulling out every container, by databasing every species and looking in parts of the collection for the first time. This might be one of the only times your collection ever gets this kind of perusal and staff will make delightful discoveries. As a result, you will know your collection better than you ever have before!

## Supplementary Material

3BCF102F-B1A3-59D8-AF93-48437CF2F812 10.3897/BDJ.8.e58310.suppl1 Supplementary material 1 Supplementary Table 1 Data type Occurrences Brief description A table of 25 largest entomology collections in the United States showing whether the collections had a species inventory and how complete it was. This information was based on Cobb et al. 2019 and Arnett 1993, the collection's website and e-mailing a contact person from the collection. File: oo_446310.xlsx https://binary.pensoft.net/file/446310 S. C. Mason, Jr., I. S. Betancourt and J. K. Gelhaus

## Figures and Tables

**Table 1.** The fourteen data entry fields in the Microsoft Excel template included in the Species Index template file, along with each field’s definition.

**DATA ENTRY FIELD**	**DEFINITION**
UNIQUE NUMBER	The unique identifier (in the form of a barcode and a human-readable number) placed on each curated “container” such as ANSP Drawers, vial racks, slide boxes, Odonata envelope boxes.
INPUT BY	Initials of the staff member entering the data (referred to as “spindexer”).
ORDER	The scientific order name. Maps to Darwin Core field “order”.
FAMILY	The scientific family name. Maps to Darwin Core field “family”.
GENUS	The scientific genus name, italicised. Maps to Darwin Core field “genus”.
SPECIES	The scientific species epithet, italicised. Maps to Darwin Core field “specificEpithet”.
SUBSPECIES	The scientific subspecies name, italicised. Maps to Darwin Core field “infraspecificEpithet”.
AUTHOR	The name of the scientist who described the species. Maps to the Darwin Core field “scientificNameAuthorship”.
NUMBER OF SPECIMENS	The exact number of specimens belonging to the species that are in the container.
PARATYPES	The exact number of specimens that have a paratype label on them.
SEE TYPE COLLECTION	A “yes” or “no” indicating if a red label is in the container to show that the primary type is represented in the Type Collection.
PRIMARY TYPE?	A “yes” or “no” indicating if a specimen could potentially be a primary type, for example, a label indicating “type”, “cotype” and “syntype."
CURATION	How the specimens are curated, for example, pinned, alcohol vial, slide.
COMMENTS	Any relevant information , for example, if a drawer were dedicated to a specific geographical region, the region would be noted.

**Table 2.** Summary of most common species epithets in the ANSP Entomology Collection. Many specimens were not identified to species and are represented by “sp.” When determinations were unable to be interpreted because of difficult handwriting and faded labels, they were recorded as “unreadable.” Specimens identified by previous entomologists as being an undescribed species were recorded as “n.sp.”

**SPECIES EPITHET**	**GENUS COUNT**
sp.	5,626
unreadable	449
*californicus* (*a*)	250
n.sp.	211
*bicolor*	198
*gracilis*	170
*occidentalis*	138
*affinis*	136
*elegans*	129
*apicalis*	122

**Table 3.** Summary of most common authors of species in the ANSP Entomology Collection. Cresson Sr. and LeConte were two of the founders of the American Entomological Society. The Society’s Collection was integrated with the Academy’s Collection in 1915.

**AUTHOR**	**SPECIES COUNT**
Cresson Sr.	3,629
LeConte	3,509
Fabricius	2,956
Say	1,869
Linnaeus	1,649
Walker	1,621
Horn	1,363
Rehn	964
Hebard	930
Dejean	896

## Hosting institution

Academy of Natural Sciences of Drexel University
